# Role of 4-1BB Receptor in the Control Played by CD8^+^ T Cells on IFN-γ Production by *Mycobacterium tuberculosis* Antigen-Specific CD4^+^ T Cells

**DOI:** 10.1371/journal.pone.0011019

**Published:** 2010-06-08

**Authors:** Carla Palma, Silvia Vendetti, Antonio Cassone

**Affiliations:** Department of Infectious, Parasitic and Immune-Mediated Diseases, Istituto Superiore di Sanità, Rome, Italy; CNRS - Université Aix-Marseille, France

## Abstract

**Background:**

Antigen-specific IFN-γ producing CD4^+^ T cells are the main mediators of protection against *Mycobacterium tuberculosis* infection both under natural conditions and following vaccination. However these cells are responsible for lung damage and poor vaccine efficacy when not tightly controlled. Discovering new tools to control nonprotective antigen-specific IFN-γ production without affecting protective IFN-γ is a challenge in tuberculosis research.

**Methods and Findings:**

Immunization with DNA encoding Ag85B, a candidate vaccine antigen of *Mycobacterium tuberculosis*, elicited in mice a low but protective CD4^+^ T cell-mediated IFN-γ response, while in mice primed with DNA and boosted with Ag85B protein a massive increase in IFN-γ response was associated with loss of protection. Both protective and non-protective Ag85B-immunization generated antigen-specific CD8^+^ T cells which suppressed IFN-γ-secreting CD4^+^ T cells. However, *ex vivo* ligation of 4-1BB, a member of TNF-receptor super-family, reduced the massive, non-protective IFN-γ responses by CD4^+^ T cells in protein-boosted mice without affecting the low protective IFN-γ-secretion in mice immunized with DNA. This selective inhibition was due to the induction of 4-1BB exclusively on CD8^+^ T cells of DNA-primed and protein-boosted mice following Ag85B protein stimulation. The 4-1BB-mediated IFN-γ inhibition did not require soluble IL-10, TGF-β, XCL-1 and MIP-1β. *In vivo* Ag85B stimulation induced 4-1BB expression on CD8^+^ T cells and *in vivo* 4-1BB ligation reduced the activation, IFN-γ production and expansion of Ag85B-specific CD4^+^ T cells of DNA-primed and protein-boosted mice.

**Conclusion/Significance:**

Antigen-specific suppressor CD8^+^ T cells are elicited through immunization with the mycobacterial antigen Ag85B. Ligation of 4-1BB receptor further enhanced their suppressive activity on IFN-γ-secreting CD4^+^ T cells. The selective expression of 4-1BB only on CD8^+^ T cells in mice developing a massive, non-protective IFN-γ response opens novel strategies for intervention in tuberculosis pathology and vaccination through T-cell co-stimulatory-based molecular targeting.

## Introduction

Tuberculosis (TB) remains a leading human infectious disease and a major public health problem in low-income countries [1]. Despite the availability of the Bacillus Calmette-Guerin (BCG) vaccine for more than 80 years, until now an effective tuberculosis vaccine is still far to be generated and still unknown are the correlates of protection against this disease [2]. There is a remarkable body of evidence that IFN-γ producing CD4^+^ T cells are the main mediators of protection against *Mycobacterium tuberculosis* (MTB) infection both under natural conditions and following vaccination [3], [4]. However, while activated CD4^+^ T cells are required to avoid the spread of MTB during infection, they can also cause severe inflammation with collateral tissue damage when not tightly controlled [5], [6]. Granuloma necrosis in a mouse model of MTB-induced pulmonary immunopathology is due to IFN-γ and T cells expressing the αβ T cell receptor [7]. Moreover IFN-γ-producing CD4^+^ T cells can interfere with development of protective immunity during experimental vaccination with mycobacterial antigens [8]–[10], including Ag85B, an abundant secreted protein of replicating MTB which is currently evaluated in various TB vaccine formulations [2], [11]–[13]. It has been recognized that activation of Ag85B-specifc CD4^+^ T cells in TB patients is not always associated with a favourable prognosis [14], [15], and that frequencies of Ag85-specific IFN-γ-secreting CD4^+^ T cells correlate with bacterial load rather than with degree of protection in MTB-infected mice [16]. The massive deleterious inflammation observed in the Koch phenomenon [17], which involves Ag85B among other antigens, is a primary example of de-regulated cell-mediated response to mycobacterial antigens. Thus, discovering how to control non protective antigen-specific IFN-γ production without affecting secretion of protective IFN-γ is an important challenge in tuberculosis research.

Regulatory T cells (Treg) limit the magnitude of effector responses. Despite the little role played by inactivation of natural CD4^+^CD25^+^ Treg cells in improving protective BCG immunity [18], several reports indicate that pathogen-specific Treg cells, mainly IL-10-secreting CD4^+^CD25^+^ Tr1, are generated following MTB infection and suppress IFN-γ cell responses in anergic TB patients [19]–[23]. A human CD8^+^LAG3^+^CD25^+^Treg subset, which can inhibit CD4^+^ T cell responses has also been described in purified protein derivative (PPD)^+^ subjects, but the clinical significance of this lymphocyte subset is still unknown [24]. The induction of Treg cells upon vaccination with mycobacterial antigen and the immunological mechanisms which regulate their functions need to be further investigated.

Co-stimulatory molecules are up-regulated by activated T cells and participate in regulation of T cell functions. 4-1BB, the murine homologue of human CD137, is a member of the TNF-receptor super-family [25] expressed primarily on antigen-receptor activated T cells [26], [27]. Although 4-1BB co-stimulates primary and secondary responses of both CD8^+^ and CD4^+^ T effector cells [28]–[31], signalling via this molecule also results in the activation of CD4^+^ Treg cells in vitro [32] and in the generation of CD8^+^ Treg cells that suppress CD4^+^ T cell function and antibody responses in vivo [33], [34]. Due to their ability to activate both effector and regulatory T cells, 4-1BB/CD137 ligands are being evaluated for treatment of cancer and auto-immune diseases [35].

We have previously reported that protection conferred by immunization of mice with an Ag85B-encoding plasmid DNA was lost when DNA-primed mice were boosted with adjuvant-free Ag85B protein [10]. The lack of protection was associated with the expansion of an Ag85B-specific CD4^+^ T cell subset secreting elevated IFN-γ amounts that caused loss or dilution of protective low IFN-γ secreting-CD4^+^ T cells elicited by DNA immunization [10]. Co-administration of Ag85B protein with the adjuvant LTK63 reduced the generation/polarization of Ag85B-specific IFN-γ secreting cells and resulted in some recovery of protection [36]. Thus, manipulating the IFN-γ production by antigen-specific CD4^+^ T cells may achieve a proper balance between protection and pathology and have vaccine-relevant implication.

In the present study, we asked whether it was possible to modulate antigen-specific, IFN-γ producing CD4^+^ T cells not in the induction phase, as mentioned above, but after they have been generated. Namely, we used the previously established TB model of protective Ag85B DNA- and non-protective Ag85B DNA/protein- immunizations to investigate the role played by CD8^+^ T cells and 4-1BB ligation on controlling memory Ag85B-specific IFN-γ-secreting CD4^+^ T cells. We found that both protective and non-protective Ag85B-immunization generated antigen-specific CD8^+^ T cells that suppressed Ag85B-specific IFN-γ-secreting CD4^+^ T cells. 4-1BB ligation selectively reduced the high but non-protective CD4^+^ T cell-mediated IFN-γ response in DNA-primed and protein-boosted mice without affecting the low IFN-γ response associated with protection in mice vaccinated with DNA. The 4-1BB-mediated IFN-γ inhibition selectively in spleen cells of DNA-primed and protein-boosted mice was linked to the induction of 4-1BB exclusively on CD8^+^ T cells of these mice upon Ag85B stimulation.

## Results

### CD8^+^ T cells reduce Ag85B-mediated IFN-γ production by antigen-specific CD4^+^ T cells elicited through protective and non-protective Ag85B-immunizations

**Figure 1:**
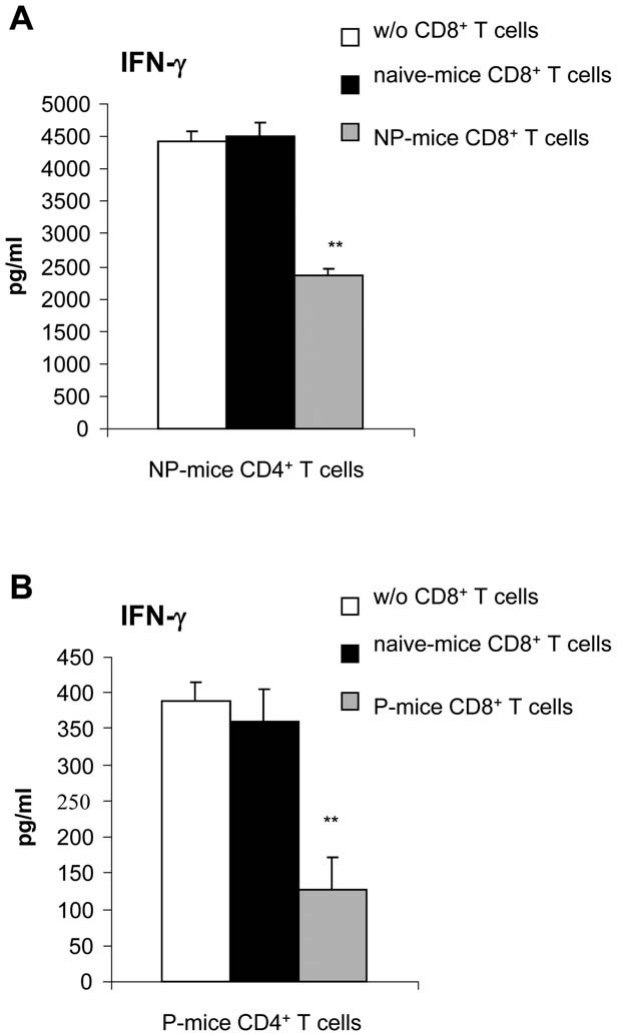
Role of CD8^+^ T cells in modulating Ag85B-mediated IFN-γ secretion associated with protection and non-protection against MTB infection. Untouched purified CD4^+^ T cells (5×10^4^ cells/well) and CD8^+^ T cells (3×10^4^ cells/well) obtained by negative magnetic bead separation as described in Materials and Methods from spleen cells of NP-mice (panel A) or P-mice (panel B) were cultured, alone or together (using the CD4/CD8 ratio of 1∶0.6 as found in fresh spleen cells) on a feeder of CD3^+^ T cell-depleted spleen cells of naive mice (1.5×10^5^ cells/well). Untouched purified CD8^+^ T cells recovered from naïve unvaccinated (3×10^4^ cells/well) were also added to CD4^+^ T cell co-culture. Cells were re-stimulated with Ag85B protein (5 microg/ml). Culture supernatants were harvested after 3 days for IFN-g detection by a specific quantitative sandwich ELISA Kit. Data are presented as mean of 4 independent experiments. Error bars indicate SEM. The level of statistical significance for differences were determined by a two-tailed Student *t* test (**, p<0.01) between Ag85B-induced responses by CD4^+^ T cells alone and co-cultured with CD8^+^ T cells. Purified CD8^+^ T cells of both naïve and Ag85B-immunized mice or cells used as feeder did not release detectable amounts of IFN-g (data not shown).

Previously, we reported that mice immunized with Ag85B-encoding DNA were protected against MTB challenge (P-mice) while adjuvant-free Ag85B protein boosting in mice primed with Ag85B-encoding DNA (NP-mice) did not confer protection (10, 36). The lack of protection in NP-mice was associated with an excessive Ag85B-induced IFN-γ production by spleen cells (10). In spleen cells of both NP- and P-mice CD4^+^ T cell depletion almost abolished the IFN-γ release indicating that IFN-γ production in response to Ag85B stimulation was mainly attributable to CD4^+^ T cells. On the other hand, depletion of CD8^+^ T cells induced a significant increase of IFN-γ secretion, suggesting that CD8^+^ T cells may exert a negative control on antigen-specific IFN-γ secreting CD4^+^ T cells (10). To verify this hypothesis, CD4^+^ and CD8^+^ T cells of NP- and P-mice were purified through negative selection and cultured on a feeder of CD3^+^ T cell-depleted spleen cells from naïve mice. CD4^+^ T cells of NP- and P-mice, (but not naïve CD4^+^ T cells used as control, data not shown) secreted IFN-γ in response to Ag85B protein stimulation maintaining the differences in magnitude observed on unfractionated spleen cells (Fig. 1 A and B). When purified CD8^+^ T cells of NP- or P-mice were added to their respective CD4^+^ T cells a significant inhibition of Ag85B-mediated IFN-γ secretion was observed (Fig. 1 A and B), indicating that CD8^+^ T cells exert suppressing activity on antigen-specific CD4^+^ T cells. Since CD8^+^ T cells recovered from naïve mice were unable to inhibit Ag85B-mediated IFN-γ secretion by CD4^+^ T cells of NP- and P- mice (Fig. 1 A and B), it is likely that these CD8^+^ T cells with suppressive activity were generated during Ag85B immunization. Purified CD8^+^ T cells of both naïve and Ag85B-immunized mice or cells used as feeder did not release detectable amounts of IFN-γ (data not shown).

### 4-1BB ligation inhibits Ag85B-mediated IFN-γ production by CD4^+^ T cells exclusively in spleen cells of NP-mice: CD8^+^ T cells are required for inhibition

Since signals through the T cell co-stimulatory molecule 4-1BB expressed by activated T cells can modulate both effector and regulatory T cell functions [28]–[34], the effects induced by 4-1BB ligation on Ag85B-mediated-IFN-γ production were studied in antigenic recall experiments with spleen cells of P- and NP-mice.

**Figure 2:**
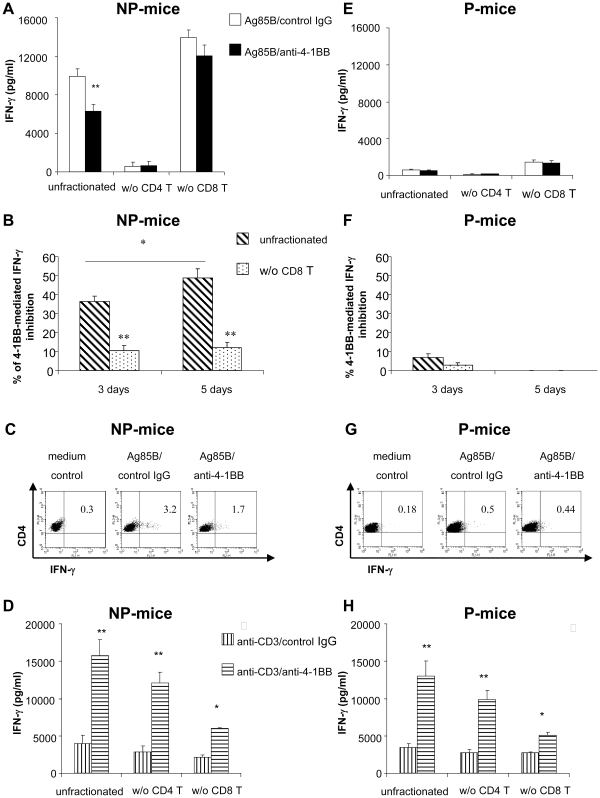
4-1BB ligation in spleen cells of NP-mice but not of P-mice reduces IFN-γ production by Ag85B-specific CD4^+^ T cells. Pooled spleen cells, unfractionated or depleted of CD4^+^ or CD8^+^ T cells by using magnetic beads as described in Materials and Methods of NP-mice (panels A–D) or P-mice (panels E–H) were re-stimulated with Ag85B protein (5 µg/ml) (panels A–C, E–G) or anti-CD3ε mAb (0.1 µg/ml) (panels D and H) in the presence of agonistic anti-4-1BB mAb or rat IgG2a control Ab (5 µg/ml each). In panels A, E, D and H, the amount of IFN-γ released in the culture supernatants after 3 days was detected by a commercial ELISA kit and the data has been reported as the mean of 5 independent experiment. In panels B and F the percentage of inhibition of Ag85B-induced IFN-γ release by an agonistic anti-4-1BB mAb in unfractionated or CD8^+^ T cell-depleted spleen population after 3 or 5 days of culture has been reported as the mean of 12 independent experiments for NP-mice and of 8 independent experiments for P-mice. Rat IgG2a control Ab did not modified the cell proliferation or IFN-γ secretion after stimulation with Ag85B and anti-CD3ε mAb (data not shown). Error bars indicate SEM. The level of statistical significance for differences between groups were determined by a two-tailed Student *t* test (*, p<0.05; **, p<0.01). In panels C and G, spleen cells of both NP- or P-mice after 4 days of culture and an overnight incubation with brefeldin A were stained with CD4-PE-Cy5 and then intracellular stained with FITC anti-IFN-γ Ab as reported in Material and Methods. Cells were then analyzed by flow cytometry. Dot plot were generated after gating on live CD4^+^ T lymphocytes and show frequency of IFN-γ-producing cells. The rat IgG2a control Ab did not significantly change Ag85B-induced IFN-γ secretion by spleen cells of NP-mice (data not shown).

In spleen cells of NP-mice stimulated with Ag85B protein, treatment with an agonistic anti-4-1BB mAb reduced the excessive/non-protective accumulation of IFN-γ in culture supernatants (Fig. 2 A and B). The lack of IFN-γ production when CD4^+^ T cell were removed from spleen cells (Fig. 2A) suggested that the 4-1BB-mediated inhibition affected the IFN-γ secretion by antigen-specific CD4^+^ T cells. This hypothesis was clearly confirmed by the consistent reduction in the percentage and fluorescence intensity of IFN-γ-producing CD4^+^ T cells in Ag85B-stimulated spleen cells treated with an agonistic anti-4-1BB mAb after 4 days of culture (Fig. 2 C and Table 1). The reduction of antigen-mediated IFN-γ accumulation in supernatants peaked at 5 days (about 48% of inhibition) (Fig. 2 B). The inhibitory effects mediated by 4-1BB ligation required CD8^+^ T cells since it was lost when those cells were removed from the cultures (Fig. 2 A and B), suggesting an involvement of the suppressor CD8^+^ T cells generated during Ag85B-vaccination in this regulatory mechanisms.

**Table 1:** Effects of 4-1BB ligation in spleen cells of NP- and P-mice stimulated with Ag85B protein on IFN-γ-producing CD4^+^ T cells and on proliferation of CD4^+^ and CD8^+^ T cells.

	CD4^+^IFN-γ^+^	CD4^+^IL-10^+^	CD4^+^CFSE^low^	CD8^+^CFSE^low^
**NP-mice**				
unstimulated	0.32±0.02	0.16±0.3	4.0±0.5	3.5±0.5
Ag85B/control IgG	5.2±0.6#	0.95±0.02#	39.8±3#	12.0±1#
Ag85B/anti-4-1BB mAb	2.6±0.3§	0.67±0.03§	24.5±0.5§	19.3±0.9§
**P-mice**				
unstimulated	0.14±0.02	nd	2.7±0.4	5.2±0.9
Ag85B/control IgG	0.62±0.06#	nd	12.3±1.9#	14.2±1.6#
Ag85B/anti-4-1BB mAb	0.4±0.13	nd	10.7±1.8	15.0±2.3

To analyze IFN-γ- or IL-10-producing CD4^+^ T cells, unfractionated spleen cells of P- or NP-mice were cultured with Ag85B protein (5 µg/ml) in the presence of IgG2a control Ab or agonist anti-4-1BB mAb (5 µg/ml). After 2 days (for IL-10 analysis) or 4 days (for IFN-γ analysis) of culture and an overnight incubation with brefeldin A cells were stained with CD4-PE-Cy5 and then intracellular stained with PE anti-IL-10 or FITC anti-IFN-γ Ab as reported in Material and Methods. Cells were then analyzed by flow cytometry and the data show frequency of IFN-γ- or IL-10-producing CD4^+^ T cells.

To study cell proliferation, unfractionated spleen cells of NP- or P-mice, were labelled with CFSE as described in Materials and Methods and then stimulated with Ag85B protein (5 µg/ml) in the presence of agonistic anti-4-1BB mAb (5 µg/ml) or rat IgG2a control Ab (5 µg/ml) for 4 days. At this time point cells were labelled with CD4-PE and CD8-PerCP and analyzed by flow cytometry as described in Materials and Methods. The percentage of CD4^+^CFSE^low^ and CD8^+^CFSE^low^ indicates the number of replicating CD4^+^ or CD8^+^ T cells, respectively.

The results are presented as mean ± SEM of the percentage of CD4^+^ T cells or CD8^+^ T cells staining for the indicated markers. Pooled data of 3 independent experiments are shown.

#, § statistical significance for differences between groups determined by ANOVA and Bonferroni-type multiple t-test (# Ag85B/control IgG vs unstimulated; § Ag85B/control IgG vs Ag85B/anti-4-1BB mAb).

nd = not done.

In spleen cells of P-mice, 4-1BB ligation did not reduce either the modest IFN-γ release by CD4^+^ T cells of P-mice stimulated with Ag85B protein - as indicated by ELISA measurement in culture supernatant of spleen cells (Fig. 2 E and F) - or the number of Ag85B-stimulated IFN-γ producing CD4^+^ T cells at 4 days of culture, as indicated by intracellular staining (Fig. 2 G and Table 1). Therefore, although suppressor CD8^+^ T cells were generated in spleens of both NP- and P-mice, 4-1BB ligation, through CD8^+^ T cells, reduced exclusively the massive, non-protective Ag85B-specific IFN-γ-production by CD4^+^ T cells of NP-mice while did not modify the modest antigenic secretion by CD4^+^ T cells of P-mice.

The capacity of 4-1BB signalling to inhibit Ag85B-mediated IFN-γ production by CD4^+^ T cells of NP-mice was antigen-restricted. Indeed, in spleen cells of both P and NP-mice, agonistic anti-4-1BB mAb treatment increased the amount of IFN-γ secreted upon activation through CD3ε engagement on T cells (Fig. 2 D and H). The up-regulation of CD3ε-mediated IFN-γ production affected both CD8^+^ (the more responsive population) and CD4^+^ T cells, as suggested by *in vitro* depletion studies (Fig. 2 D and H). Therefore, 4-1BB acted as the expected co-stimulatory molecule in view of its ability to enhance IFN-γ production by polyclonally-activated CD4^+^ and CD8^+^ T cells.

### Ag85B stimulation induces expression of 4-1BB on CD8^+^ T cells exclusively in spleen cells of NP-mice

**Figure 3:**
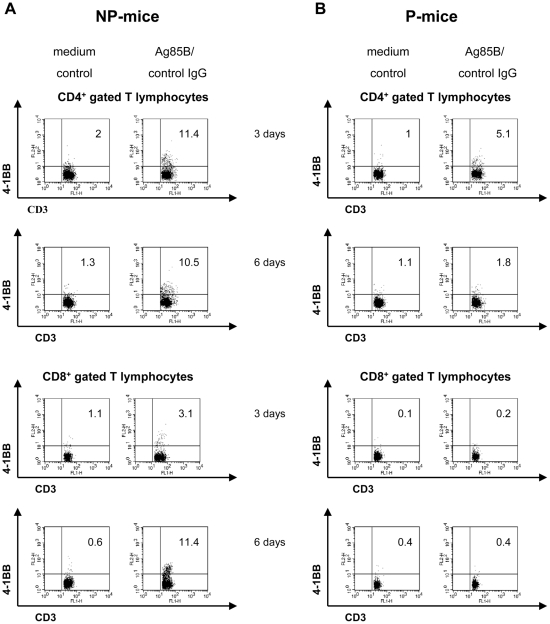
Ag85B protein stimulation in spleen cells of NP-mice induces expression of 4-1BB on both CD4^+^ and CD8^+^ T cells. Pooled spleen cells of NP-mice (panel A) or P-mice (panel B) were re-stimulated with Ag85B protein (5 µg/ml). After 3 or 6 days of cultures, cells were labelled with the following mAb: CD4 PE-Cy7, CD8 PE-Cy7, CD3 FITC and 4-1BB PE. Cell surface molecule expression was analyzed by flow cytometry as described in Materials and Methods. Within each panel, the percentage indicates the number of cells expressing the 4-1BB receptor. Plots, gated on CD4^+^ or CD8^+^ T cells in the lymphocyte-gate, are representative of one experiments out of 3.

In order to explain the responsiveness of spleen cells of NP-mice to 4-1BB ligation, we analyzed whether Ag85B stimulation could induce the expression of this TNF-receptor on T cells. In spleen cells of NP-mice induction of 4-1BB expression was found on a sub-population of CD4^+^ T cells when splenocytes were stimulated for 3 days with Ag85B protein (Fig. 3 A and Table 2). 4-1BB expression on CD4^+^ T cells was maintained after 6 days of culture. In spleen cells of P-mice activated with Ag85B protein a smaller percentage of CD4^+^ T cells, as compared to Ag85B-activated CD4^+^ T cells of NP-mice, expressed 4-1BB after 3 days of culture. However, after 6 days of culture no significant expression of this receptor was found on CD4^+^ T cells of P-mice (Fig. 3 A and Table 2).

**Table 2:** Effects of 4-1BB ligation in spleen cells of NP- and P-mice stimulated with Ag85B protein on expression of 4-1BB receptor on CD4^+^ and CD8^+^ T cells.

	3 days		6 days	
	CD4^+^4-1BB^+^	CD8^+^4-1BB^+^	CD4^+^4-1BB^+^	CD8^+^4-1BB^+^
**NP-mice**				
unstimulated	1.5±0.5	1.1±0.05	2.3±0.3	1.7±0.4
Ag85B	11.7±0.3**	3.5±0.5**	13.4±1.3**	11.5±1.7**
**P-mice**				
unstimulated	1.05±0.05	0.2±0.1	1.3±0.13	0.35±0.05
Ag85B	4.5±0.6**	0.2±0.1	3.1±0.7	0.45±0.05

Unfractionated spleen cells of P- or NP-mice were cultured with Ag85B protein (5 µg/ml). After 3 or 6 days of culture cells were labeled with the following labelled mAb: CD4 PE-Cy7, CD8 PE-Cy7, CD3 FITC and 4-1BB PE. Cell surface molecule expression was analyzed by flow cytometry as described in Materials and Methods. The results are presented as mean ± SEM of the percentage of CD4^+^ T cells or CD8^+^ T cells positive for 4-1BB. Pooled data of 3 independent experiments are shown.

**, p<0.01, statistical significance for differences between groups Ag85B vs unstimulated determined by a two-tailed Student's t-test.

In spleen cells of NP-mice Ag85B protein stimulation caused the induction of 4-1BB on a small subset of CD8^+^ T cells after 3 days of culture and the percentage of 4-1BB^+^CD8^+^ T cells even increased after 6 days of culture (Fig. 3 B and Table 2). Therefore, in response to antigen, some CD4^+^ and CD8^+^ T cells of NP-mice became responsive to ligands for 4-1BB. On the other hand, no significant induction of 4-1BB was found on CD8^+^ T cells in spleen cells of P-mice activated with Ag85B protein after 3 or 6 days of culture (Fig. 3 B and Table 2).

### Effect of 4-1BB ligation on modulation of CD4^+^ and CD8^+^ T cell surface marker expression in Ag85B-activated spleen cells of NP mice

Next, the effects of 4-1BB ligation on the phenotype of both CD4^+^ and CD8^+^ T cells was studied in culture of spleen cells of NP- or P- mice stimulated with Ag85B protein. In particular, the expression of T cell activation markers expressed upon TCR complex stimulation such as CD25, the IL-2Rα, CD38, an ectoenzyme with cyclase and hydrolase enzymatic activity, CD233, an MHC class II binding CD4 homologue, and CD44, an adhesion molecule expressed by activated memory T cells, was analyzed. All these markers are up-regulated by both activated effector and regulatory cells [21], [23], [32]–[34], [37]–[41].

In spleen cells of NP-mice Ag85B protein stimulation induced up-regulation of CD25, CD38, CD233 and CD44 on about 40% of CD4^+^ T cells (Table 3). Likely these CD4^+^ T cells were effectory memory cells in view of the low expression of CD62L (data not shown). Upon agonistic anti-4-1BB mAb treatment, CD4^+^ T cells of NP-mice down-modulated surface expression of all activated T cell markers, suggesting a reduced activation of antigen-specific CD4^+^ T cells following agonistic anti-4-1BB mAb treatment.

**Table 3:** Effects of 4-1BB ligation in spleen cells of NP- and P-mice stimulated with Ag85B protein on expression of activation markers on CD4^+^ T cells.

		CD4^+^ gated T	lymphocytes	
	CD25	CD38	CD233	CD44^high^
**NP-mice**				
unstimulated	14.0±2.0	17.0±0.6	10.0±1.0	15.5±0.5
Ag85B/control IgG	40.5±0.5#	44.6±1.3#	49±3.0#	41.5±0.5#
Ag85B/anti-4-1BB mAb	30.5±1.5§	29.6±0.9§	35.5±1.5§	26.5±0.5§
**P-mice**				
unstimulated	10.6±0.6	16.0±0.1	5.5±0.5	14.0±0.1
Ag85B/control IgG	20.7±0.6#	24.6±0.3#	14.5±0.5#	21.0±1.0#
Ag85B/anti-4-1BB mAb	23.0±1.0	25.0±1.7	14.0±1.0	22.5±0.5

Unfractionated spleen cells of P- or NP-mice were cultured with Ag85B protein (5 µg/ml) in the presence of IgG2a control Ab or agonist anti-4-1BB mAb (5 µg/ml). After 6 days of culture cells were labeled with the indicated mAbs. Cell surface molecule expression was analyzed by flow cytometry as described in Materials and Methods. The results are presented as mean ± SEM of the percentage of CD4^+^ T cells staining for the indicated markers. Pooled data of 3 independent experiments are shown.

#, § statistical significance (p<0.05 or <0.01) for differences between groups determined by ANOVA and Bonferroni-type multiple t-test (# Ag85B/control IgG vs unstimulated; § Ag85B/control IgG vs Ag85B/anti-4-1BB mAb).

In spleen cells of P-mice, Ag85B stimulation induced a weak up-regulation of CD25, CD38, CD233 and CD44 expression on CD4^+^ T cells (Table 3). These data were consistent with a reduced activation of CD4^+^ T cells of P-mice compared to CD4^+^ T cells of NP-mice upon Ag85B protein stimulation. Moreover, agonistic anti-4-1BB mAb treatment did not influence the phenotype of Ag85B-activated CD4^+^ T cells.

In spleen cells of both P- and NP-mice Ag85B protein stimulation led to up-regulation of CD233 on a subset of CD8^+^ T cells, while no changes were observed in the percentage of cells expressing CD25, CD38 and CD44 (Table 4). Agonistic anti-4-1BB mAb treatment induced down-modulation of CD233 exclusively on CD8^+^ T cells of NP-mice.

**Table 4:** Effects of 4-1BB ligation in spleen cells of NP- and P-mice stimulated with Ag85B protein on expression of activation markers on CD8^+^ T cells.

		CD8^+^ gated T	lymphocytes	
	CD25	CD38	CD233	CD44^high^
**NP-mice**				
unstimulated	10.5±1.5	12.4±1.0	14.4±1.2	7.3±1.4
Ag85B/control IgG	9.6±2.1	13.3±1.0	32.8±1.1#	8.6±1.6
Ag85B/anti-4-1BB mAb	9±1.2	13.1±1.0	22.4±1.3§	12.5±1.2
**P-mice**				
unstimulated	8.0±1.5	15.5±1.6	6.0±1.0	9.7±0.8
Ag85B/control IgG	6.6±1.2	15.5±0.2	15.3±0.3#	7.7±0.9
Ag85B/anti-4-1BB mAb	6.7±0.8	16±1.0	15.5±0.5	8.9±1.0

Unfractionated spleen cells of P- or NP-mice were cultured with Ag85B protein (5 µg/ml) in the presence of IgG2a control Ab or agonist anti-4-1BB mAb (5 µg/ml). After 6 days of culture cells were labeled with the indicated mAbs. Cell surface molecule expression was analyzed by flow cytometry as described in Materials and Methods. The results are presented as mean ± SEM of the percentage of CD8^+^ T cells staining for the indicated markers. Pooled data of 3 independent experiments are shown.

#, § statistical significance (p<0.05 or <0.01) for differences between groups determined by ANOVA and Bonferroni-type multiple t-test (# Ag85B/control IgG vs unstimulated; § Ag85B/control IgG vs Ag85B/anti-4-1BB mAb).

### 4-1BB ligation has opposite effects on cell proliferation in spleen of NP-mice stimulated with Ag85B: reducing proliferation of CD4^+^ T cells while enhancing that of CD8^+^ T cells

In order to analyze the effects of 4-1BB ligation on CD4^+^ and CD8^+^ T cell proliferation of NP- and P-mice, the CFSE labelled spleen cells were stimulated with Ag85B protein for 4 days.

**Figure 4:**
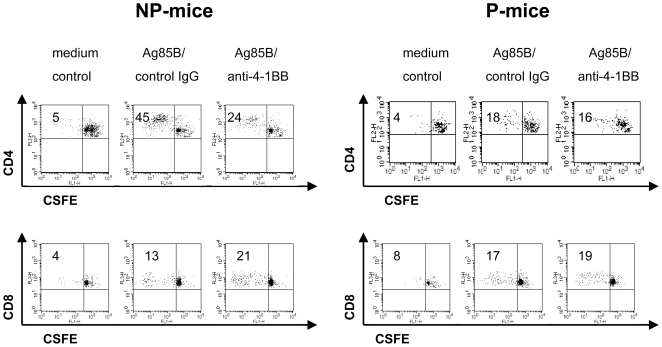
Effects of 4-1BB ligation in spleen cells of NP-mice stimulated with Ag85B protein on CD4^+^ and CD8^+^ T cell proliferation. Pooled spleen cells of NP- or P-mice, were labelled with CFSE as described in Materials and Methods and then stimulated with Ag85B protein (5 µg/ml) in the presence of agonistic anti-4-1BB mAb (5 µg/ml) or rat IgG2a control Ab (5 µg/ml) for 4 days. At this time point cells were labelled with CD4-PE and CD8-PerCP and analyzed by flow cytometry as described in Materials and Methods. The percentage indicates the number of replicating CD4^+^ or CD8^+^ T cells. Plots, gated on CD4^+^ or CD8^+^ T cell in the lymphocyte-gate, are representative of one experiments out of 3.

In spleen cells of NP-mice, Ag85B protein stimulation induced a remarkable CD4^+^ T cell proliferation as indicated by CFSE dilution (Fig. 4 and Table 1). Treatment with an agonistic anti-4-1BB mAb significantly reduced the number of proliferating CD4^+^ T cells (Fig. 4 and Table 1). These data are consistent with the reduced expression of activation markers observed on Ag85B-activated CD4^+^ T cells of NP-mice following 4-1BB ligation (Table 3). Ag85B protein stimulation also induced CD8^+^ T cell proliferation. However, the number of proliferating CD8^+^ T cells increased in the presence of the agonistic anti-4-1BB mAb treatment, suggesting that the subset of CD8^+^ T cells expressing 4-1BB may be expanded by a direct engagement of this receptor (Fig. 4 and Table 1).

In spleen cells of P-mice, Ag85B-stimulation induced a lower CD4^+^ T cell proliferation as compared to that observed in CD4^+^ T cells of NP-mice while the number of proliferating CD8^+^ T cells were similar in the two groups of immunized mice (Fig. 4 and Table 1). Agonistic anti-4-1BB mAb treatment had only a little influence on CD4^+^ or CD8^+^ T cell proliferation in P-mice (Fig. 4 and Table 1). These data are consistent with the low or absent expression of 4-1BB on CD4^+^ T and CD8^+^ T cells of P-mice.

### Effects of 4-1BB ligation on cytokine/chemokine production in Ag85B-stimulated spleen cells of NP-mice

**Figure 5:**
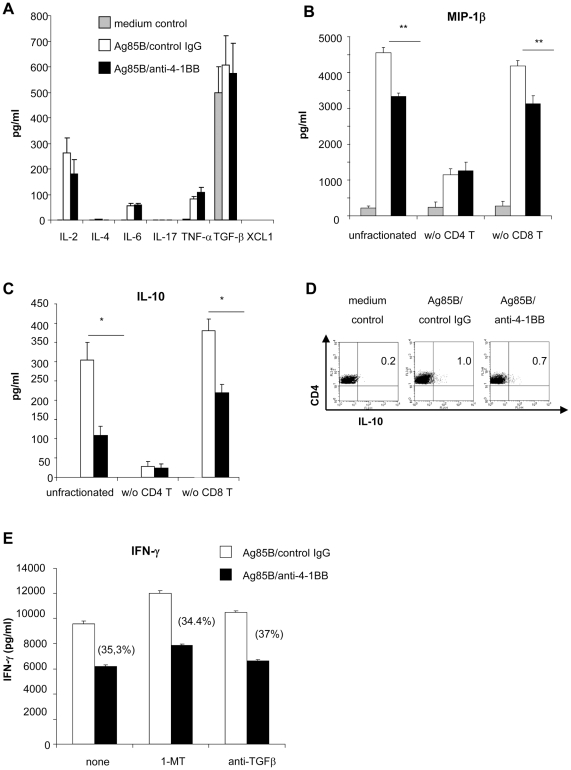
Effects of 4-1BB ligation in spleen cells of NP-mice stimulated with Ag85B protein on cytokine/chemokine production. Pooled spleen cells of NP-mice, unfractionated or depleted of CD4^+^ or CD8^+^ T cells by using magnetic beads as described in Materials and Methods (panels B and C), were re-stimulated with Ag85B protein (5 µg/ml) in the presence of agonistic anti-4-1BB mAb (5 µg/ml) or rat IgG2a control Ab (5 µg/ml). Culture supernatants were harvested at 48 h for IL-2, IL-4, at 72 h for IFN-γ, IL-6, IL-10, MIP-1β, TGF-β1, TNF-α, XCL-1 and at 96 h for IL-17 detection by specific quantitative sandwich ELISA Kits. Data are presented as mean of 3 independent experiments for panels A, B, and C. Error bars indicate SEM. The level of statistical significance for differences between Ag85B stimulation in the presence of IgG2a control Ab or agonistic anti-4-1BB mAb were determined by a two-tailed Student *t* test (*, p<0.05; **, p<0.01). In panel D, spleen cells after 2 days of culture and an overnight incubation with brefeldin A were stained with CD4-PE-Cy5 and then intracellular stained with PE anti-IL-10 Ab as reported in Material and Methods. Cells were then analyzed by flow cytometry. Dot plot were generated after gating on live CD4^+^ T lymphocytes and show frequency of IL-10-producing cells. In panel E, the IDO-inhibitor, 1-MT (400 µM), or neutralizing anti-TGF-β1.2 mAb (2 µg/ml) were added simultaneously to culture of spleen cells stimulated with Ag85B protein (5 µg/ml) in the presence of agonistic anti-4-1BB mAb (5 µg/ml) or rat IgG2a control Ab (5 µg/ml). Culture supernatants were harvested at 72 h for IFN-γ detection by specific quantitative sandwich ELISA Kits. Percent of 4-1BB-mediated inhibition of Ag85B-induced IFN-γ release is shown in round brackets.

We next asked whether 4-1BB signalling could modulate Ag85B-mediated production of other cytokines, besides IFN-γ, playing a recognized positive or negative role in protection against MTB. Cytokine/chemokines such IL-10, TGF-β, MIP-1β and XCL-1 capable to inhibit the IFN-γ production by CD4^+^ T cells [24], [42]–[44] were also analyzed to address the mechanisms of 4-1BB-mediated IFN-γ inhibition. In spleen cells of NP-mice, the low Ag85B-induced secretion of IL-2 and TNF-α, Th1 associate cytokines was preserved in the presence of agonist anti-4-1BB mAb (Fig. 5 A). Also IL-6 release induced by Ag85B protein stimulation was unaffected by agonist anti-4-1BB mAb treatment (Fig. 5 A). 4-1BB ligation did not induce production of IL-4, a Th2 cytokine, IL-17, a cytokine which contribute to protection to MTB challenge [45], [46] and XCL-1, a chemokine released by suppressor CD8^+^ T cells during the chronic stage of infection with MTB that negatively affects production of IFN-γ by CD4^+^ T cells [44]. None of the cytokines above were released upon Ag85B stimulation (Fig. 5 A). Although no further secreted in response to antigenic stimulation, some TGF-β, a potent cytokine suppressing Th1 function [38], was nonetheless found in spleen cell cultures (Fig. 5 A). Hence, to exclude any involvement of TGF-β in 4-1BB-mediated inhibition of IFN-γ, an anti-TGFβ neutralizing mAb was added to unfractionated spleen cell culture of NP-mice stimulated with Ag85B protein and agonistic anti-4-1BB mAb. Also in this condition, the ability of 4-1BB ligation to reduce Ag85B-induced IFN-γ secretion was unchanged (Fig. 5 E).

On the other hand, 4-1BB ligation decreased the accumulation of IL-10 and MIP-1β (Fig. 5 B and C). Since soluble forms of IL-10 and MIP-1β can both control IFN-γ production [19], [23], [24], [42] the reduced level of these factors found following 4-1BB ligation did not fit with a their involvement in 4-1BB-mediated IFN-γ inhibition. IL-10 was exclusively produced by CD4^+^ T cells upon Ag85B stimulation in total spleen cells (Fig. 5 C and D) and 4-1BB-ligation reduced IL-10 production by Ag85B-stimulated CD4^+^ T cells as shown in intracellular staining (Fig. 5 D). IL-10-producing CD4^+^ T cells did not produce IFN-γ (data not shown), indicating that in spleen cell populations two different Ag85B-responding CD4^+^ T cell subsets secreting IL-10 or IFN-γ were present. Since secretion of MIP-1β was in part due to cells other than CD4^+^T-cells (Fig. 5 B), the possibility of MIP-1β secretion by CD8^+^ T cells was supposed. In any case 4-1BB ligation did not modulate the release of MIP-1β by non-CD4^+^ T cells (Fig. 5 B).

Moreover, unlike IFN-γ, CD4^+^ T cell-mediated production of IL-10 and MIP-1β decreased in a CD8^+^ T cell-independent manner upon 4-1BB ligation (Fig. 5 B and C), suggesting that in Ag85B-responding CD4^+^ T cells the regulatory mechanisms controlling secretion of IFN-γ differ compare to those modulating other cytokines influential in TB protection.

In addition to cytokines, some metabolic products which affect tryptophan metabolism play a role in inhibition of T cell responses. In particular, the tryptophan catabolising enzyme indoleamine-pyrrole 2,3 dioxygenase (IDO) leads to the inhibition of CD4^+^ T cell responses when up-regulated by an agonistic 4-1BB mAb in macrophages and dendritic cells [34], [47]. To test IDO functions we used 1-methyltryptophan (1-MT), which abrogates 4-1BB-controlled IDO-mediated immune suppression [47]. When 1-MT was added to unfractionated spleen cell culture of NP-mice, the ability of 4-1BB ligation to reduce Ag85B-induced IFN-γ secretion was unchanged (Fig. 5 E). However, 1-MT treatment induced increased IFN-γ levels in Ag85B-stimulated cells independent of treatment with an agonistic anti-4-1BB mAb (Fig. 5 E). This was presumably caused by the high level of IFN-γ in culture, since IFN-γ up-regulates IDO in macrophages and dendritic cells (34).

In spleen cells of P-mice, treatment with the agonistic anti-4-1BB mAb did not significantly modify Ag85B-induced release of IL-2, IL-6, MIP-1β and TNF-α, similarly to IFN-γ production, and did not induce release of IL-10, IL-4, TGF-β, IL-17 and XCL-1 (Table 5), supporting again the unresponsiveness of these T cells to ligands for 4-1BB.

**Table 5:** Effects of 4-1BB ligation in Ag85B-activated spleen cells of P-mice on cytokine/chemokine production.

	medium control	Ag85B/control IgG	Ag85B/anti-4-1BB mAb
IL-2	0±0	144±60	97±50
IL-4	0±0	0±0	0±0
IL-6	0±0	41±14	37±17
IL-10	0±0	0±0	0±0
IL-17	0±0	0±0	0±0
TNF-α	0±0	32±6	44±6
MIP-1 β	30±20	2436±195	2770±140
TGF−β1	713±102	702±98	768±130
XCL-1	0±0	0±0	0±0

Unfractionated spleen cells of P-mice were cultured with Ag85B protein (5 µg/ml) in the presence of IgG2a control Ab or agonist anti-4-1BB mAb (5 µg/ml). Culture supernatants were harvested at 48 h for IL-2, IL-4; at 72 h for IL-6, IL-10, XCL-1, MIP-1β, TGF-β1, TNF-α and at 96 h for IL-17 detection by specific quantitative sandwich ELISA Kits. The results are presented as mean ± SEM in pg/ml for cytokine detection. Pooled data of 3 independent experiments are shown.

### 4-1BB ligation on purified CD4^+^ T cells of NP-mice stimulated with Ag85B protein inhibits release of IL-10 and MIP-1β, but not IFN-γ: requirement of CD8^+^ T cells for 4-1BB-mediated IFN-γ inhibition

**Figure 6:**
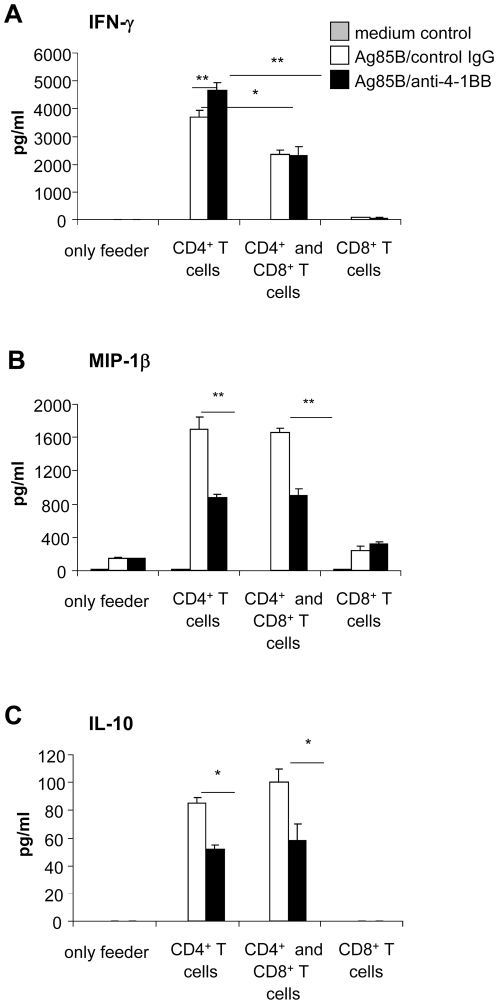
4-1BB ligation on purified CD4^+^ T cells of NP-mice inhibits Ag85B-induced release of IL-10 and MIP-1β, but not IFN-γ: requirement of CD8^+^ T cells for 4-1BB-mediated IFN-γ inhibition. Untouched purified CD4^+^ T cells (5×10^4^ cells/well) and CD8^+^ T cells (3×10^4^ cells/well) obtained by negative magnetic bead separation as described in Materials and Methods from spleen cells of NP-mice were cultured, alone or together (using the CD4/CD8 ratio of 1∶0.6 as found in fresh spleen cells) on a feeder of CD3^+^ T cell-depleted spleen cells of naive mice (1.5×10^5^ cells/well). Cells were re-stimulated with Ag85B protein (5 µg/ml) in the presence of agonistic anti-4-1BB mAb or rat IgG2a control Ab (5 µg/ml each). Culture supernatants were harvested at at 72 h for IFN-γ, (panel A), MIP-1β (panel B) and IL-10 (panel C) detection by specific quantitative sandwich ELISA Kits. Data are presented as mean of 4 independent experiments. Error bars indicate SEM. The level of statistical significance for differences were determined by a two-tailed Student's *t*-test (*, p<0.05; **, p<0.01) between Ag85B stimulation in the presence of rat IgG2a control Ab or agonistic anti-4-1BB mAb (panels A–C) and between Ag85B-induced responses (both in the presence or absence of agonist anti-4-1BB mAb) by CD4^+^ T cells alone and co-cultured with CD8^+^ T cells ( panel A).

Since Ag85B-stimulated CD4^+^ T cells of NP-mice expressed the 4-1BB molecule, the effects induced by a direct engagement of this TNF-receptor on these cells were investigated. Therefore, antigenic recall studies were performed on purified CD4^+^ T cells cultured on a feeder of CD3^+^ T cell-depleted spleen cells of naive mice. In addition to IFN-γ (as previously shown in Fig. 1), IL-10 and MIP-1β were also secreted in such co-culture upon Ag85B protein stimulation (Fig. 6 A–C). Treatment with an agonistic anti-4-1BB mAb, while increasing IFN-γ release, significantly reduced CD4^+^ T cell-mediated-secretion of IL-10 and MIP-1β (Fig. 6 A–C).The effects induced by 4-1BB ligation on release of these cytokines were attributable to CD4^+^ T cells since CD3^+^ T cell-depleted spleen cells of naive mice used as feeder did not release IFN-γ and IL-10 in response to Ag85B protein and/or to agonistic anti-4-1BB mAb, and released only a low amount of MIP-1β not modified by 4-1BB treatment (Fig. 6 A–C). As previously shown in Fig. 1A, addition of purified CD8^+^ T cells of NP-mice to the co-culture significantly reduced IFN-γ secretion by Ag85B-stimulated CD4^+^ T cells (Fig. 6 A). Treatment of these CD4^+^/CD8^+^ T cell-co-culture with the agonistic anti-4-1BB mAb resulted in a further IFN-γ inhibition (50.2±3.5 versus 36±3 in the presence or absence of 4-1BB ligation, respectively) (Fig. 6 A), suggesting that 4-1BB ligation on 4-1BB^+^CD8^+^ T cells enhanced the suppressive effects exerted by CD8^+^ T cells on CD4^+^ T cell-mediated IFN-γ release. A low, almost undetectable release of IFN-γ by purified CD8^+^ T cells was found upon Ag85B stimulation, and IFN-γ secretion was not significantly modified by 4-1BB ligation, further confirming that IFN-γ released by spleen cells upon antigenic recall was mainly due to CD4^+^ T cells. Differently from what observed for IFN-γ release, the presence of CD8^+^ T cells neither affected release of IL-10 and MIP-1β by Ag85B-stimulated CD4^+^ T cells nor modified the reduction of IL-10 and MIP-1β secretion caused by a direct engagement of 4-1BB on Ag85B-activated CD4^+^ T cells (Fig. 6 B and C). Purified CD8^+^ T cells released low amount of MIP-1β upon Ag85B stimulation that remained unaltered following 4-1BB ligation while IL-10 was undetectable in CD8^+^ T cell culture supernatants (Fig. 6 B and C).

Antigenic recall experiments performed with purified CD4^+^ and/or CD8^+^ T cells of P-mice indicated that treatment with agonistic anti-4-1BB mAb did not influence secretion of IFN-γ and MIP-1β by CD4^+^ T cells nor the CD8^+^T cell-mediated inhibition of IFN-γ release by CD4^+^ T cells (data not shown). These results are in agreement with the lack o low 4-1BB expression on Ag85B-stimulated T cells of P-mice.

### In vivo antigenic stimulation induces expression of 4-1BB on Ag85B-specific CD8^+^ T cells and 4-1BB ligation inhibits the antigen-mediated IFN-γ production and expansion of CD4^+^ T cells from NP-mice

**Figure 7:**
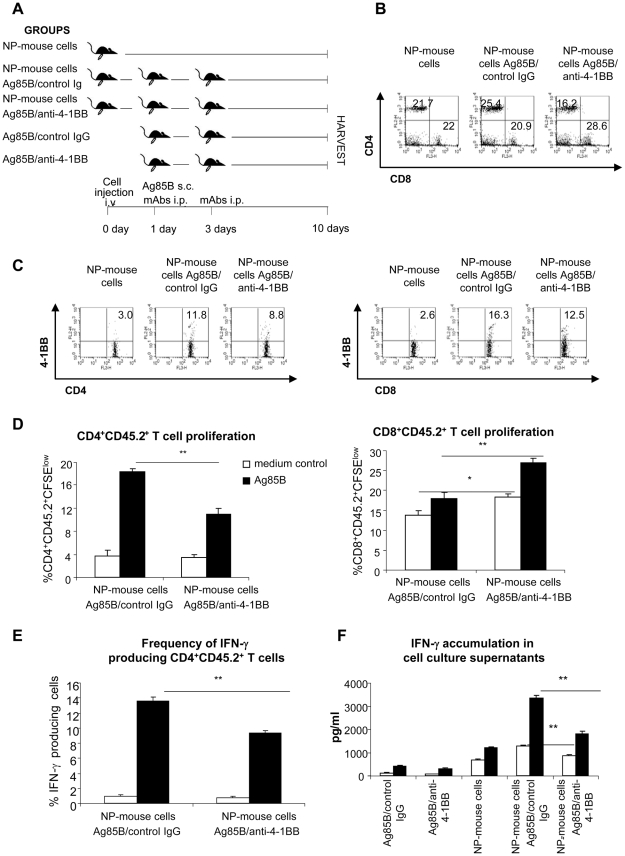
Effects of *in vivo* Ag85B stimulation and 4-1BB ligation on CD4^+^ and CD8^+^ T cells of NP-mice. Spleen cells from NP-mice were adoptively transferred into C57BL/6Ly5.1 recipient mice accordingly to the scheme shown in panel A. 10^7^ splenocytes from NP-mice (CD45.2^+^ cells) were adoptively transferred i.v. into C57BL/6Ly5.1 recipient mice (expressing CD45.1 leukocyte antigen). On p.i. day 1, mice were inoculated s.c. with 10 µg of recombinant Ag85B protein and then i.p. with 150 µg agonistic anti-mouse 4-1BB mAb or with 150 µg rat IgG2a isotype control mAb. On p.i. day 3, mice were again injected with 150 µg agonistic anti-mouse 4-1BB mAb or with 150 µg rat IgG2a isotype control Ab. As controls, a group of mice adoptively transferred with splenocytes from NP-mice was treated with neither antigen Ag85B nor mAbs, and two groups of mice, not inoculated with splenocytes from NP-mice, received only the treatment with Ag85B and mAbs as descrived above. On p.i. day 10, fresh spleen cell recovered from mice adoptively transferred with NP-mice and treated *in vivo* with Ag85B and mAb were labelled with CD45.2-FITC, CD4-PE, CD8-PerCP, 4-1BB-PE, CD4-PerCPCy5.5 and analyzed by flow cytometry. In panel B, the percentage indicates the frequency of CD4^+^ and CD8^+^ T cells originating from NP-mice and in panel C the percentage of those CD4^+^ and CD8^+^ T cells expressing 4-1BB. Plots, gated on CD45.2^+^ cells in the lymphocyte-gate, are representative of 1 out of 3 mice per group. Spleen cells of C57BL/6Ly5.1 were then used for antigenic recall *ex vivo* experiments. Cells were re-stimulated *ex vivo* with Ag85B protein (5 µg/ml). In panel D spleen cells, labelled with CFSE at day 0 as described in Materials and Methods, after 4 days of culture were stained with CD4-PE, CD8-PerCP and CD45.2-APC and analyzed by flow cytometry. The percentage indicates the number of replicating CD4^+^CD45.2^+^or CD8^+^CD45.2^+^ T cells. In panel E the frequency of IFN-γ-producing CD4^+^CD45.2^+^ T cells were measured by FACS analysis after 4 days of culture and an overnight incubation with brefeldin A on cells labelled with CD4-PerCP-Cy5.5, CD45.2-FITC and then intracellular stained with PE anti-IFN-γ Ab. Cells were then analyzed by flow cytometry. In panel F the amount of IFN-γ released in the culture supernatants after 4 days was detected by a commercial ELISA kit. The data has been reported as the mean of 3 individual determinations for each group. Error bars indicate SEM. The level of statistical significance for differences between unstimulated and *ex vivo* antigen-stimulated spleen cells of mice adoptively transferred with cells from NP-mice and treated *in vivo* with Ag85B protein in the presence of IgG2a control Ab or agonistic anti-4-1BB mAb were determined by a two-tailed Student *t* test (*, p<0.05; **, p<0.01).

To evaluate the effects of *in vivo* 4-1BB triggering on Ag85B-specific CD4^+^ and CD8^+^ T cells, adoptive transfer experiments with spleen cells of NP-mice were performed. Spleen cells from NP-mice (CD45.2^+^) were injected into C57BL/6Ly5.1 recipient mice (expressing CD45.1 leukocyte antigen). On post-injection (p.i.) day 1, mice were inoculated s.c. with 10 µg of recombinant Ag85B protein and then i.p. with 150 µg agonistic anti-mouse 4-1BB mAb or with 150 µg rat IgG2a isotype control mAb. On p.i. day 3, mice were again injected with 150 µg agonistic anti-mouse 4-1BB mAb or with 150 µg rat IgG2a isotype control Ab. As controls, a group of mice adoptively transferred with splenocytes from NP-mice was treated with neither antigen Ag85B nor mAbs, and two groups of mice, not inoculated with splenocytes from NP-mice, received only the treatment with Ag85B and mAbs as descrived above. On p.i. day 10, all mice were sacrificed and spleens were harvested for immunological studies (Fig. 7 A).

In spleen cells of mice adoptively transferred with cells from NP-mouse the frequency of CD4^+^ and CD8^+^ T cells originating from NP-mice following antigenic stimulation *in vivo* with Ag85B protein in the presence of anti-4-1BB mAb or control IgG2a mAbs was analyzed by gating lymphocytes positive for CD45.2. We found that injection of Ag85B into mice induced a significant expansion of Ag85B-specific CD4^+^ T cells (Fig. 7 B, Table 6). Moreover, *in vivo* Ag85B treatment, induced the expression of 4-1BB on both CD4^+^ and CD8^+^ T cells (Fig. 7 C, Table 6), suggesting that *in vivo* 4-1BB engagement could modulate their responses. In fact, treatment with anti-4-1BB mAb *in vivo* significantly reduced the percentage of CD4^+^ T cells responding to Ag85B treatment. Although the increase of CD8^+^ T cell frequency was not statistically significant, *in vivo* 4-1BB ligation significantly reversed the ratio of CD4^+^ and CD8^+^ T cells (0.64±0.3 vs 1.2±0.01 in mice injected with Ag85B and anti-4-1BB mAb compared with mice injected with Ag85B and control IgG2a mAb, respectively (Fig. 7 B, Table 6). This indicates an expansion of CD8^+^ T cells at the expense of CD4^+^ T cells in mice treated with anti-4-1BB mAb. To better clarify whether *in vivo* 4-1BB ligation downgrades the action of Ag85B-specific CD4^+^ T cells while expanding CD8^+^ T cells, we also performed *ex vivo* antigenic recall experiments with spleen cells of mice adoptively transferred with cells from NP-mouse. After 4 days of culture the percentage of proliferating CD8^+^CD45.2^+^T cells, both in unstimulated and *ex vivo* Ag85B-restimulated splenocytes, was significantly enhanced in anti-4-1BB mAb-treated mice (Fig. 7 D). On the contrary, the anti-4-1BB mAb *in vivo* treatment significantly reduced the frequency of proliferating CD4^+^CD45.2^+^T cells (Fig. 7 D) as well as the percentage of IFN-γ-producing CD4^+^CD45.2^+^T cells responding to *ex vivo* antigenic re-stimulation (Fig. 7 E). Moreover, IFN-γ accumulation in culture supernatants, both in unstimulated and re-stimulated ex vivo with Ag85B protein spleen cell culture, was significantly reduced in anti-4-1BB mAb-treated mice (Fig. 7 F). The IFN-γ released in the cultures was mainly released by cells originating from NP-mice (Fig. 7 F). Altogether these data indicate that *in vivo* 4-1BB ligation reduced antigen-mediated-activation of Ag85B-specific CD4^+^ T cells while expanding CD8^+^ T cells of NP-mice, in keeping with the results of the *in vitro* experiments (see above).

**Table 6:** Effects of Ag85B stimulation and 4-1BB ligation on in vivo frequency of CD4^+^ and CD8^+^ T cells and 4-1BB expression of cells from NP-mice adoptively transferred into C57BL/6Ly5.1 recipient mice.

		CD45.2^+^ gated	T lymphocytes	
Treatments			CD4^+^4-1BB^+^	CD8^+^4-1BB^+^
	CD4^+^ T cells	CD8^+^ T cells	T cells	T cells
none	21.2±0.5	21.8±0.5	2.8±0.1	2.4±0.1
Ag85B/control IgG	25.8±0.3#	22.1±0.8	10.1±1.0#	15.4±0.9#
Ag85B/anti-4-1BB mAb	16.7±0.5§	25.8±1.3	7.1±0.6§	12.3±0.8

C57BL/6Ly5.1 mice adoptively transferred with 10^7^ spleen cells from NP-mice (cells CD45.2^+^) were treated or not with antigen Ag85B and mAbs as described in Materials and Methods. Slpeen cells were harvested on post-infection day 10, stained with CD45.2-FITC, CD4-PE, CD8-PerCP, CD4PerCPCy5.5 and 4-1BB-PE mAbs and then analyzed by flow cytometry. The data, obtained by gating CD45.2^+^ cells on lymphocyte population, show the frequency of CD4^+^ and CD8^+^ T cells originating from NP-mice and the percentage of T cells expressing 4-1BB. The results are presented as mean ± SEM of 3 mice per group.

#, § statistical significance for differences between groups determined by ANOVA and Bonferroni-type multiple t-test (# Ag85B/control IgG vs none; § Ag85B/control IgG vs Ag85B/anti-4-1BB mAb).

## Discussion

Dampening the drawbacks of elevated antigen-specific IFN-γ secretion by CD4^+^ T cells without affecting the protective IFN-γ response is a difficult challenge in TB research, particularly to generate a strongly immunogenic though safe vaccine. In a murine model of TB, combined DNA/protein immunization with the mycobacterial antigen Ag85B (NP-mice) increases IFN-γ production by antigen-specific CD4^+^ T cells but reduces protection from challenge relative to the marked protection seen in mice immunized with DNA alone (P-mice) (10, 36). Here we report that both protective and non-protective Ag85B-immunizations generate antigen-specific CD8^+^ T cells which, in *ex vivo* experiments, suppress IFN-γ-production by Ag85B-specific CD4^+^ T cells. Signals via the TNF-receptor 4-1BB down-modulate, via CD8^+^ T cells, the high-level of IFN-γ production by Ag85B-specific CD4^+^ T cell associated with poor protection in NP-mice while preserving the low IFN-γ response that is associated with protective immunity in P-mice. The observed inhibition of memory IFN-γ-secreting CD4^+^ T cell in NP-mice was likely due to the induction, upon Ag85B stimulation, of 4-1BB expression selectively on suppressor CD8^+^ T cells of NP-mice. Thus, the 4-1BB capability of discriminating between low and high antigen-specific CD4^+^ T-cell-mediated IFN-γ responses could be instrumental to find novel strategies based on T cells and co-stimulatory signals to down-regulate the effects of elevated antigen-specific IFN-γ responses associated with TB pathology and its negative impact on vaccine protection. As a matter of fact, knowledge of T-cell co-stimulatory molecules on MTB immunity and their potential use as new therapeutic targets is still lacking. In addition, *in vivo* 4-1BB ligation reduced the activation, IFN-γ production and expansion of CD4^+^ T cells of NP-mice re-stimulated *in vivo* with Ag85B, suggesting that the inhibitory phenomenon observed *in vitro* can also occur *in vivo*.

CD8^+^ T cells have a recognized, direct role in protection against TB through their cytotoxic activity on MTB-infected cells [48]. The main finding of this paper strongly indicate that CD8^+^ T cells may have an additional role in TB as regulator of CD4^+^ T cell-mediated responses, which are mainly implicated in protection against MTB infection [3], [4]. A role for CD8^+^ Treg cells was postulated to explain increased IFN-γ production by antigen-specific murine CD4^+^ T cells following CD8^+^T cell-depletion *ex vivo* [10] and in vivo [44]. Here we provide the first evidence of antigen-induced suppressor CD8^+^ T cells generated in vivo that inhibit CD4^+^ T cell proliferation and IFN-γ secretion *ex vivo*. These suppressor CD8^+^ T cells were found in the spleen of both NP- and P-mice suggesting that they are an intrinsic component of the response to immunization. Indeed, splenic CD8^+^ T cells of naive mice did not show any regulatory activity. Moreover, while suppressor CD8^+^ T cells were generated both in P- and NP-mice, 4-1BB ligation suppressed the CD4^+^ T cell-mediated IFN-γ response only in NP-mice. This suppressive effect was linked to the induction, upon Ag85B protein stimulation, of 4-1BB receptor expression only on CD8^+^ T cells of NP-mice. Thus, CD8^+^ T cells of NP-mice responded to 4-1BB ligands while CD8^+^ T cells of P-mice were insensitive to 4-1BB ligation because they did not express 4-1BB molecules. Consistent with these results is the enhancement of antigen-specific cell proliferation of CD8^+^ T cells of NP-mice but not of P-mice following agonistic anti-4-1BB mAb treatment.

The elevated level of IFN-γ found in spleen cell cultures of NP-mice upon Ag85B protein stimulation may be responsible for the selective expression of 4-1BB on the surface of CD8^+^ T cells of NP-mice. In fact, during chronic inflammation, 4-1BB receptor expression was up-regulated on CD8^+^ T cell surface by inflammatory mediators including IFN-γ [49], [50]. Therefore expression of 4-1BB on suppressor CD8^+^ T cells in mice producing elevated amount of IFN-γ may be a self-regulatory mechanism that curtails excessive, antigen-driven IFN-γ response in MTB infection. This hypothesis is in agreement with the high expression of 4-1BB induced by Ag85B stimulation *in vivo* on CD8^+^ T cells of NP-mice and expansion of these cells upon *in vivo* anti-4-1BB mAb treatment. The role played by 4-1BB in regulating CD8^+^ Treg cells deserves to be further investigated in TB and also in other infectious diseases. In fact, induction of pathogen-specific CD8^+^ Treg cells, involved in regulating both pathogen eradication and immunopathology [51], [52], have been described in various bacterial [53], viral [54]–[56] and parasitic infections [57].

The molecular mechanisms underlying 4-1BB mediated restriction of IFN-γ production remain to be elucidated. Apparently, none of the mechanisms thus far proposed to explain inhibition of IFN-γ secreting CD4^+^ T cells applies to our findings. First, soluble MIP-1β secreted by CD8^+^CD233^+^CD25^+^ Treg has been proposed to suppress antigen-mediated CD4^+^ T cell responses in PPD^+^ responders via inhibition of Ca^2+^ flux [24]. Even though we found release of MIP-1β by CD8^+^ T cells upon Ag85B stimulation and 4-1BB ligation, the inhibitory action of soluble MIP-1β did not match MIP-1β release by Ag85B-activated CD4^+^ T cells nor the reduced, total MIP-1β accumulation in spleen cells upon 4-1BB ligation. In addition, the high CD38 expression on CD4^+^ T cells might have counteracted MIP-1β inhibition of Ca^2+^ flux by activating Ca^2+^ influx through an ADPR-gated cation channel [37]. Second, release of IL-10 and TGF-β, which are main mediators of CD8^+^ and CD4^+^ Treg cells [42], [43], [51] are not relevant for suppression of IFN-γ production in our experiments. Agonist anti-4-1BB mAb did not increase the basal TGF-β accumulation found in spleen cultures and a neutralizing anti-TGF-β mAb was ineffective in contrasting 4-1BB effects on IFN-γ release. 4-1BB engagement did not induce secretion of IL-10 by CD8^+^ T cells, and even down-regulated the secretion of IL-10 by CD4^+^ T cells. Taking into account that IL-10-secreting CD4^+^ Treg isolated from TB patients have been often considered a poor prognostic factor [19]–[23], the IL-10-independent mechanisms of IFN-γ regulation described in this paper may constitute a protective mechanism that limits dangerous inflammation without being associated with the anergy induced by IL-10 in TB patients. Third, XCL-1, a chemokine produced by activated CD8^+^ T cells during the chronic stage of MTB infection, which negatively affects production of IFN-γ by CD4^+^ T cells [44], was not released by CD8^+^ T cells of NP-mice either upon Ag85B stimulation or following 4-1BB ligation. Fourth, the inability of an IDO inhibitor to counteract 4-1BB regulatory effects indicate that IDO accumulation in macrophages and dendritic cells - a mechanism which suppress CD4^+^ T cell responses in experimental uveoretinitis and rheumatoid arthritis through release of IFN-γ by 4-1BB-activated T cells [34], [47] - was not activated by Ag85B-specific CD8^+^ T cells and was not involved in control of Ag85B-induced IFN-γ response.

The adoptive transfer experiments clearly demonstrated that *in vivo* treatment with anti-4-1BB mAb inhibited antigen-mediated activation, IFN-γ production and expansion of Ag85B-specific CD4^+^ T cells of NP-mice. Although the cellular and molecular mechanisms operating *in vivo* remain to be elucidated, an at least partial involvement of CD8^+^ T cells is suggested by the antigen-mediated expression of 4-1BB on CD8^+^ T cells, and by their proliferation following 4-1BB ligation *in vivo*. Interestingly, it has been recently reported that 4-1BB ligation *in vivo* expanded a subset of CD8^+^ T cells involved in suppression of the recall response to staphylococcal enterotoxin A-specific CD4^+^ T cells [58].

Besides inhibition of IFN-γ production, agonist anti-4-1BB mAb treatment of splenocytes of NP-mice induced several changes in Ag85B-specific CD4^+^ T cells consistent with reduced cellular activation. Cell proliferation was lowered, expression of activation marker such as CD38, CD25, CD44 and CD233 was down-modulated, and secretion of MIP-1β and IL-10 was also decreased. The 4-1BB-mediated de-activation of Ag85B-specific CD4^+^ T cells occurred via CD8^+^ T suppressor cells only for IFN-γ modulation while inhibition of IL-10 and MIP-1β secretion was attributable to a direct triggering of 4-1BB on surface of CD4^+^ T cells. Altogether these data indicate that in NP-mice, Ag85B-responding CD4^+^ T cells are a non-homogeneous population or they are regulated by distinct pathways which are differentially affected by 4-1BB ligation. Thus, in mycobacterial antigen-specific CD4^+^ T cells, 4-1BB treatment reduced the release of excessive and dangerous IFN-γ, inhibited secretion of IL-10 and MIP-1β, which are often associated with TB susceptibility [19], [59] while preserved the low production of IL-2 and TNF-α, two cytokines which are essential for granuloma maintenance [60], [61] and are expressed, together with IFN-γ, in polyfunctional T cells associated with protection from MTB infection [62].

Overall, the novel regulatory effects shown by agonistic 4-1BB ligands on modulation of potentially dangerous responses of CD4^+^ T cells specific for Ag85B - an antigen actively secreted by replicating MTB and proposed as a candidate sub-unit TB vaccine - open novel strategies for intervention in TB pathology and vaccination through T-cell co-stimulatory-based molecular targeting. Although the role played by 4-1BB *in vivo* during MTB infection needs further investigations, this receptor could be effectively involved in the immunological responses against mycobacterial infection since expression of CD137, the human counterpart of 4-1BB, was found on a sub-population of the T-lymphocytes infiltrating lung tuberculous granulomas in patients with TB [63].

## Materials and Methods

### Ethics Statement

The handling of mice were conducted in accordance with the regulations set forward by the institutional animal care committee of the Italian Ministry of Health, and in compliance with European Community Directive 86/609 and the U.S. Association for Laboratory Animal Care recommendations for the care and use of laboratory animals.

### Immunizations

C57BL/6 female mice were supplied as specific pathogen-free mice by Harlan (Udine, Italy) and were maintained in specific-pathogen-free conditions. Mice, 6 to 8 week old, were immunized. Fifty µg of plasmid Ag85B-encoding DNA, prepared as previously reported [10], was injected i.m. in 50 µl PBS into the hind leg. On the dorsum of the mice, 10 µg of recombinant Ag85B protein, prepared as previously reported [10], was co-administered s.c. Recombinant Ag85B protein was free of physiologically significant levels of endotoxin, as measured by LAL system. Mice were immunized twice with Ag85B-encoding DNA at 2-week intervals (P-mice). Some DNA-primed-mice were boosted with Ag85B protein twice at 2-week intervals (NP-mice). Four weeks after the last immunization, spleens were harvested for immunological studies.

### Spleen cell preparation and separation of lymphocytes

Single cell suspensions from pooled spleens (4 mice per group, 4 separate experiments) were applied to Falcon 2360 cell strainers (BD Discovery Labware, San Diego, CA), centrifuged, separated into aliquots and frozen. From spleen cell suspension, isolated CD4^+^ or CD8^+^ T cells and the corresponding CD4- or CD8-depleted spleen cell populations, were purified by negative selection using specific mouse CD4^+^ or CD8^+^ T cell isolation kits (Miltenyi Biotec Inc. Auburn, CA), in accordance with manufacturer's instructions. The purity of isolated CD4^+^ or CD8^+^ T cells ranged between 94% and 96% as estimated by FACS analysis.

### Spleen cell cultures

Cells isolated from spleens of immunized mice, either unfractionated or CD4- and CD8-depleted, were cultured at a density 1.4×10^6^cells/ml in 96-well plates for cell proliferation and cytokine detection or in 24-well plates for cytokine detection and flow cytometry analysis in RPMI-1640 supplemented with 10% heat-inactivated FBS, 2 mM L-glutamine, 10 mM HEPES buffer, 50µM 2-β-ME, 50 U/ml penicillin and 50 µg/ml streptomycin (complete RPMI, cRPMI). Cells were stimulated with 5 µg/ml Ag85B protein or 0.1 µg/ml agonistic anti-mouse CD3ε mAb (BD Pharmingen, San Jose, CA) in the presence or absence of 5 µg/ml agonistic anti-mouse 4-1BB/TNFRSF9/CD137 mAb (clone 158321) (R&D System, Inc. Minneapolis, MN) or rat IgG2a isotype control Ab (clone 544479) (R&D System, Inc). In some culture, the IDO-inhibitor, 1-methyltryptophan (1-MT) (Sigma-Aldrich, Saint Louis, MO) 400 µM or neutralizing anti-TGFβ1 mAb (clone 9016) (R&D System, Inc.) were also added.

In other experiments, untouched purified CD4^+^ or CD8^+^ T cells were magnetically isolated from the specific mouse CD4^+^ or CD8^+^ T cell isolation kits, (Miltenyi Biotec), in accordance with manufacturer's instructions. Purified untouched CD4^+^ T cells (5×10^4^ cells/well) or purified untouched CD8^+^ T cells (3×10^4^ cells/well) were cultured on a feeder of spleen cells recovered from naïve unvaccinated mice and depleted of CD3^+^ T lymphocyte by a magnetic separation through a PE-isolation kit (Miltenyi Biotec) (1.5×10^5^ cells/well). Co-cultures of purified untouched CD4^+^ and CD8^+^ T cells were performed at a CD4/CD8 ratio of 1∶0.6, which reflected the CD4/CD8 ratio observed by FACS analysis in fresh splenocytes. Cells were stimulated with 5 µg/ml Ag85B protein with or without 5 µg/ml agonistic anti-mouse 4-1BB mAb or 5 µg/ml rat IgG2a control Ab.

### Cytokine detection in culture supernatants

Supernatants of cultured cells were harvested at 2, 3, 4 and 5 days and assayed for IFN-γ, IL-10, IL-4, IL-2, IL-6, IL-17, TNF-α, TGF-β, MIP-1β and XCL-1 by specific quantitative sandwich ELISA (mouse Quantikine, R&D System, Inc. or RayBiotech, Inc. Norcross, GA), in accordance with manufacturer's instructions.

### Flow cytometric analysis

Fresh spleen cells or cells cultured in 24 well plates for 4 or 6 days, were washed with 1% BSA, 0.1% NaN_3_ in PBS 1× (FACS buffer), and stained for 20 min at 4°C with the following mAbs: PE-Cy5 anti-mouse CD4, PerCPCy5.5 anti-mouse CD4, PE-Cy7 anti-mouse CD8 (eBioscience, San Diego CA), FITC anti-mouse CD11c (Serotec LTD, Oxford, UK), PerCP anti-mouse CD8, FITC anti-mouse CD3, PE anti-mouse 4-1BB, FITC anti-mouse CD38, FITC anti-mouse CD25, PE anti-mouse CD233, FITC anti-mouse CD44, PE anti-mouse CD62L and FITC anti-mouse CD45.2 (BD Biosciences Pharmingen). Cells were washed and analyzed on a FACScan flow cytometer (Beckton Dickinson Immunocytometry System, San Jose, CA) with the Cell Quest software program. Staining of samples with isotype controls was used as a reference to determine positive and negative populations. For intracellular cytokine staining, spleen cells were treated overnight with 10 µg/ml of brefeldin A (Sigma-Aldrich), an inhibitor of protein transport from endoplasmic reticulum to the Golgi apparatus. Then cells were stained for 20 min at 4°C with PE-Cy5 anti-mouse CD4, PerCPCy5.5 anti-mouse CD4, FITC anti-mouse CD45.2 or PercPE anti-mouse CD8, washed and fixed for 5 min with 4% paraformaldehyde. Cells were washed and stained with FITC- anti-mouse IFN-γ, PE-anti-mouse IFN-γ, PE anti-mouse IL-10 or isotype controls (BD Biosciences Pharmingen) in FACS buffer with 0.5% saponin for 45 min at R.T. Cells were then washed in FACS buffer with 0.1% saponin and subsequently with FACS buffer with 0.01% saponin and then analyzed on a FACScan flow cytometer with the Cell Quest software program.

### Cell proliferation by CFSE staining

Spleen cells (10^7^ cells/ml) were stained with 5(6)-Carboxyfluorescein diacetate N-succinimidyl ester (CFSE) (Invitrogen Life Technologies) at 1 µM in PBS 1%FBS for 10 min at 37°C in the dark. Cells were washed and cultured in 96-well plates, as previously described, for 4 days. After the incubation time, cells were washed with FACS buffer, and stained for 20 min at 4°C with PE anti-mouse CD4, PerCP anti-mouse CD8, APC anti-mouse CD45.2 (BD Biosciences Pharmingen) or the isotype controls. Cells were washed and analyzed on a FACScan flow cytometer with the Cell Quest or DIVA software programs.

### Adoptive transfer experiments

10^7^ splenocytes from NP-mice (about 1.53×10^6^ CD4^+^T cells and 1.16×10^6^ CD8^+^T cells; these T cells are CD45.2^+^) were adoptively transferred intravenously via caudal vein injection into C57BL/6Ly5.1 recipient mice (same strain of mice expressing CD45.1 instead of CD45.2 on their leukocytes). On postinjection (p.i.) day 1, mice (3 mice each group) were inoculated s.c. with 10 µg of recombinant Ag85B protein and then i.p. with 150 µg agonistic anti-mouse 4-1BB/TNFRSF9/CD137 mAb (clone 158321) or with 150 µg rat IgG2a isotype control mAb (clone 544479). On p.i. day 3, mice were again injected with 150 µg agonistic anti-mouse 4-1BB mAb or with 150 µg rat IgG2a isotype control Ab. As controls, a group of mice adoptively transferred with splenocytes from NP-mice was treated with neither antigen Ag85B nor mAbs, and two groups of mice, not inoculated with splenocytes from NP-mice, received only the treatment with Ag85B and mAbs as descrived above. On p.i. day 10, all mice were sacrificed and spleens were harvested for immunological studies.
